# Green finance, renewable energy development, and climate change: evidence from regions of China

**DOI:** 10.1057/s41599-023-01595-0

**Published:** 2023-03-15

**Authors:** Yunpeng Sun, Qun Bao, Farhad Taghizadeh-Hesary

**Affiliations:** 1 School of Economics, Tianjin University of Commerce, Tianjin, China; 2 School of Economics, Nankai University, Tianjin, China; 3 School of Global Studies, Tokai University, Tokyo, Japan

**Keywords:** Economics, Finance

## Abstract

In this study, using data from 2010 to 2021, and by utilizing the stochastic impacts by regression on population, affluence, and technology (STIRPAT) theory, and system generalized method of moments, the effect of green financing and deployment of renewable energy on carbon dioxide emissions in China and its provinces were analyzed. The results show that green financing reduces environmental pollution at the country level. Moreover, with a 1% increase in renewable energy consumption, carbon dioxide emission can be expected to decrease by 0.103%. It also demonstrates that green financing has a statistically significant coefficient only in provinces located in the eastern and western regions. Chinese policymakers should incentive policies for provinces in the eastern region of China in order to have a cleaner environment. The central region should be under supportive and pressure policies to move faster along the path to sustainable development.

## Introduction

Climate change is a complex concept in the literature on environmental economics and human challenge in the global context. Undoubtedly, climate change causes disturbances in agricultural production (risk to food security), global warming, droughts, and geopolitical tensions related to water. Some scholars believe that the threat of climate change is unsolvable in the short term because of its global nature; therefore, it requires global unity and policy to be solved. Li et al. (2021) have argued that climate change has no simple solution and necessitates global unity and alignment. Froehlich et al. (2022) have stated that the threat of climate change is a potential risk to food security, economic resilience, and global peace. Therefore, there is an urgent need for more international efforts to eliminate this threat.

However, it should be noted that efforts have been made, albeit small and integrated, to deal with climate change. These efforts (e.g., the U.N. Framework Convention on Climate Change of 1992, the Kyoto Protocol of 1992, and the Paris Agreement of 2015) have included international agreements to implement integrated and aligned policies to reduce the threat of climate change. Huang and Zhai (2021) have mentioned that global treaties are inefficient on their own, and countries’ commitment to these agreements must increase. Zhang et al. (2017) have highlighted the importance of countries’ alignment to make international agreements on climate change more efficient. They have mentioned the withdrawal of the U.S. from the Paris Agreement, which has lowered the validity of this global treaty. Other efforts have included regional cooperation and a plan to overcome the challenges of climate change. In this regard, the Regional Dialog on Carbon Pricing in ASEAN, the 2030 E.U. Climate Target Plan, and the Africa Clean Energy Corridor can be considered successful cases. Rabe (2022) has declared that regional planning to mitigate climate change threats can be considered a significant step toward global unification. In another study, Das et al. (2022) have confirmed that intra-regional planning for renewable energy sources can efficiently promote environmental protection and combat climate change. Other efforts have included local policies to promote environmental protection, green energy deployment, and lower carbon emissions.

Countries have implemented various policies to combat climate change. As a roadmap for implementing such policies, countries must have a long-term plan or clear vision of their sustainability goals. For example, in 2020, China announced its plans to achieve peak carbon by 2030 and carbon neutrality by 2060; in 2020, Japan declared its plan to implement net-zero emissions by 2050. Other plans have included CanREA’s 2050 Vision, UAE’s National Energy Strategy 2050, Qatar’s National Vision 2030, Turkish renewable energy vision 2023, Korea’s Energy Master Plan 2035, Germany’s landmark Renewable Energy Act 2021, and Singapore’s Green Plan 2030; all of these roadmaps have clear targets and set out intermediate, operational, and final goals.

Under these long-term plans, countries are implementing deterrent and incentive policies and tools based on their economies’ systems, weaknesses, and strengths. The most important policies are listed in Table 1.

**Table 1:** Countries’ policies to combat the climate change threat.

Policy	Case of implementation	Source
Green loan policy	China, the UAE	Zhang (2022), Al-Qudah et al. (2022), Tan et al. (2022)
Green tax policy	Iran, Germany, China, Ecuador	Norouzi et al. (2022), van der Ploeg et al. (2022), Wang and Yu (2021), Terneus Páez et al. (2022)
Green bonds policy	The E.U., China, Japan, the U.S.	Cicchiello et al. (2022), Li et al. (2022), Han and Li (2022), Tolliver et al. (2020)
Green innovation policy	China, E7	Wang et al. (2022), Hao and Chen (2022)
Green job creation policy	Italy, the U.S., China	Bowen et al. (2018), Zhang and Du (2020), Dell’Anna (2021)
Green transportation	China, the UAE, Pakistan	Kiani (2017), Meng et al. (2021), Shahid et al. (2022)

Source: The authors.

The critical point for implementing the policies listed in Table 1 is the development of related projects and sufficient capital to invest in them. Therefore, financing green projects has been one of the most critical and challenging topics among experts (e.g., Tolliver et al., 2020; see also Cicchiello et al., 2022, Li et al., 2022; Han and Li, 2022) in recent years. Without an efficient green financing system, a country cannot advance its sustainable development goals or reduce climate-related threats. Green finance can be seen as a bridge to renewable energy deployment in countries, particularly where the government does not have sufficient capital to invest in green projects. Rasoulinezhad and Taghizadeh-Hesary (2022) have declared that green finance is a helpful instrument for governments that cannot invest significantly in renewable energy projects. On the one hand, green financing can increase the number of green projects implemented in countries; on the other hand, it will attract more capital from the private sector to green projects.

This study primarily examines the relationship between green finance, renewable energy deployment, and climate change in China. China is a leading Asian country in terms of establishing various plans to develop green energy consumption across various industries. As shown in Table 1, China is among the countries that have implemented policies (green tax, green loans, green financing, green innovation, green jobs, and green transportation) to diversify pathways to reach its sustainability targets. In the last decade, financing green projects has become a priority for the Chinese government. According to The State Council of China (2021), the country provided over 2.5 trillion U.S. dollars in green loans by the end of 2021 (nearly 30% growth over 2020). It should be mentioned that in 2020, China ranked first in the world in providing green loans (USD 1.8 trillion) and second in the world in issuing green bonds (USD 125 billion). The development of green financial policies in China has become more critical after its vision to achieve peak carbon by 2030 and carbon neutrality by 2060. Wang et al. (2021) have emphasized the role of China’s two current climate change policies (peak carbon and carbon neutrality) as a pathway toward the era of the Chinese climate economy. Therefore, China should attempt to make its green financial system more efficient and attract more private capital to green economic projects.

Given the importance of studying the case of China regarding how green finance and renewable energy deployment can affect climate change, this research makes the following contributions to the existing literature:

i. It conducts a regional study of China that does not consider China’s country-level data. China has more than 1.4 billion people and a relatively wide geographical area (9,600,013 sq. km). Therefore, it should be regionally investigated. Therefore, in this study, data analysis of different regions of China was conducted as a panel data framework.
ii. It uses the stochastic impacts by regression on population, affluence, and technology (STIRPAT) theoretical framework to explain the research problem and econometric modeling.
iii. It uses carbon dioxide emissions per capita as a proxy for climate change and issued green bonds as a proxy for the green financing variable.

The results showed that green financing reduces environmental pollution at the country level. Additionally, with a 1% increase in renewable energy consumption, carbon dioxide emission can be expected to decrease by 0.103%. Therefore, green financing has a statistically significant coefficient only in provinces located in the eastern and western regions. Chinese policymakers should institute incentive policies in provinces in the eastern region of China. The central region should be under supportive and pressure policies to move faster along the path to sustainable development.

The remainder of this paper proceeds as follows. Section “Literature review” provides a brief literature review and clarifies the literature gaps this study tries to fill. The section “Green policies in China” provides information on green policies in China. Section “Data and model specification” discusses the data and model specifications, and the section “Empirical results” presents the empirical findings. The last section presents the concluding remarks, a discussion of practical policies, and recommendations for further research.

## Literature review

Several earlier studies have drawn attention to the impacts of green finance and renewable energy deployment on climate change in China.

A group of scholars has focused on the importance of green finance in China’s sustainable development. Li et al. (2021) used the wavelet power spectrum approach to investigate the role of green finance in China after the COVID-19 pandemic. The major findings have confirmed the unidirectional causal relationship between renewable energy and green finance in China. In a recent study, Zhou and Xu (2022) evaluated the impact of green finance on regional ecological enhancement in China. The results of the estimations using the generalized method of moments (GMM) model revealed a U-shaped relationship between the two variables. Zhu et al. (2022) have discussed the role of green finance in terms of the development goals in China. Analyzing annual data from 1986 to 2019, they revealed a positive association between green finance and green development in China. Irfan et al. (2022) have investigated the relationship between green finance and green innovation at the regional level in China. Using the panel Vector Autoregression (VAR) technique and annual data from 2010 to 2019, they showed that green finance positively impacts green innovation across all regions of China. Lin et al. (2022) have explored the impact of green finance on CO_2_ emissions reduction in China from 2007 to 2018. A dynamic spatial Durbin model has confirmed that green finance in China is efficient and can lower carbon dioxide emissions. Zhang et al. (2022) attempted to determine how green finance enhanced urban city-level green development in China from 2002 to 2019. The major results highlighted the role of green finance in lowering urban energy intensity. Hou et al. (2022) concentrated on five provinces in China in 2017 to explore the role of green financial policies in promoting environmental quality. They employed a difference-in-differences model and concluded that green finance has a heterogeneous effect among the examined provinces.

Previous studies have focused on the impact of green energy deployment on climate change in China. Qi et al. (2014) have attempted to identify a link between CO_2_ emissions and renewable energy development in China from 2010 to 2020. The major findings confirmed the positive role of renewable power generation in reducing CO_2_ emissions in the short term. Chen et al. (2019) employed the autoregressive distributed lag (ARDL) bounds testing approach using annual data from 1980 to 2014 to study the relationship between renewable energy and CO_2_ emissions in China. They have concluded that any increase in green energy deployment leads to decreased carbon dioxide emissions in the short and long term. Recently, Wang (2022) used the ARDL approach to analyze annual data from 2007 to 2019 in China to determine how green energy can help the country achieve its decarbonization target. The results showed that, in the long term, green energy development could decrease carbon dioxide emissions in China. Dong et al. (2022) employed the STIRPAT approach to study carbon emissions mitigation in China. They have found a negative correlation between green energy deployment and carbon emissions. Abbasi et al. (2022) compared the impacts of green and fossil fuel energy resources on climate change in China. Employing dynamic ARDL and annual data from 1980 to 2018, they have concluded that green energy deployment has short- and long-term positive impacts on CO_2_ mitigation. In another study, Lin and Qiao (2022) explored how green electricity can be adopted into people’s lifestyles to help China lower its environmental pollution. The major results revealed that reasonably priced green electricity could stimulate people to use it, leading to lower carbon emissions in China. Zhu et al. (2022) have addressed the role of green energy resources in mitigating fossil-fuel-based CO_2_ emissions in rural China from 2007 to 2018. The findings have highlighted the significant long-term impact of green energy resource development on rural environmental protection in the country. Wang et al. (2023) studied net energy-related carbon dioxide emissions in China. The empirical findings have confirmed the positive role of green energy development in industrial production in the country. Zhang et al. (2023) also confirmed the positive effect of green energy resources on agricultural land expansion in China. Farmland expansion policy can be strengthened through the deployment of renewable energy sources.

Based on a summary of the most recent research, it can be said that many studies have been conducted on green finance in China; however, it seems that the results of such research are not useful to policymakers. Hou et al. (2022) have shown that the impact of green financing on climate threats in China is heterogeneous across different provinces. Additionally, the STIRPAT method, as a supporting theory of the econometric model, has rarely been used (see Dong et al., 2022). The selection of variables for the econometric model should have a clear theoretical basis. This study considered the effects of renewable energy consumption and green financing on China’s climate threat using a model. Furthermore, the selection of modeling variables was explained based on the structure of the STIRPAT theory. The analysis was conducted at the regional level in China. Each of the aforementioned cases represents a literature gap that this research will fill.

## Green policies in China

The history of green policies in China dates back to 1972 when China attended the first United Nations Conference on the Human Environment. A decade later, in 1983, the Chinese government announced that protecting the country’s environment required government policy. In 1998, the State Environmental Protection Administration (SEPA) was established because of a flood disaster that became a national crisis. From its establishment in 1998 to 2002, the current administration has carried out essential actions in the environmental field. The SEPA policy of converting fields into forests in China during this period caused more than 7.7 million hectares of agricultural land to be converted to forest land. From 2002 to 2015, various green policies were developed in China, most of which were ineffective because of the need for fossil fuels to support China’s manufacturing industries. The signing of the Paris Agreement on December 12, 2015, by China and 195 other countries set a suitable road map for China to pay more attention to environmental issues. Additionally, the introduction of the Sustainable Development Goals by the United Nations on January 1, 2016, doubled China’s motivation to achieve sustainable development. In April 2019, China promoted the Belt and Road Initiative International Green Development Coalition as the first Chinese regional sustainable plan. In 2020, China launched its vision of peak carbon by 2030 and carbon neutrality by 2060 as two major projects aimed at improving air quality and environmental protection in the global context. China is a pioneer in implementing green financing to support green projects. Since March 2016, according to China’s 13th Five-Year Plan for Economic and Social Development, the country has established a green financing system that includes green credit, green bonds, and green development funds. The Agricultural Bank of China issued its first green bonds in late 2015. Recently, the Chinese government has supported these policies. In 2021, China’s green bond market was the second largest after that of the U.S. The country issued over 1643 green bonds valued at USD 270 billion in 2021. Regarding green loans, Chinese banks provided nearly USD 2.5 trillion in 2021, compared to around 1.8 trillion in 2020. China’s economic sector targets for developing green financing markets are green transportation, emerging industries, and industrial energy savings. In February 2021, the Chinese State Council published a roadmap for the transition pathway toward green and recycling development by 2025, and enhanced environmental protection in pursuit of the target of a “beautiful China” by 2035 (China briefing, 2022). In February 2022, the Chinese State Council announced guidelines to accelerate the establishment and enhancement of a green and renewable economic system.

## Theoretical background

In this study, the supporting theory of econometric modeling is the STIRPAT theory, presented by Dietz and Rosa (1997). Given that this study aims to evaluate the impacts of green finance and renewable energy development on climate change, the effects of the variables on climate change could be addressed through the popular environmental STIRPAT pattern. This theory addresses the impact of human activity (I) on the quality of the environment. Human activity (I) is comprised of the following three factors: population (P), affluence (A), and technology (T). The advantages of employing this theory to construct the empirical model are as follows: (i) this theory can explain the channels of impacts of issued green bonds and renewable energy deployment on climate change; (ii) this theory is useful for hypothesis decomposition, which is necessary to obtain practical policies for countries; and (iii) anthropogenic factors such as urbanization, human development, and health can be evaluated through STIRPAT theory. This directly impacts the relationship between human activity and the environment.

The relationship between the environment and human activity in the STIRPAT framework can be represented by Eq. (1) as follows:

1 $$I = f\left( {P,A,T} \right)$$

In Eq. (1), carbon dioxide emissions per capita are used as a proxy for the impact of climate change, whereas *P*, *A*, and *T* denote population (labor force as proxy), affluence (income per capita as proxy), and technology proxy with green finance.

## Data and model specification

According to the theoretical framework, Eq. (1) can be transformed into an econometric Eq. (2), as follows:

2 $$I_{i,t} = \eta P_{i,t}^{\varphi _1}A_{i,t}^{\varphi _2}T_{i,t}^{\varphi _3}\mu _{i,t}$$

Equation (2) in the logarithm form can be transformed into Eq. (3), as follows:

3 $${\rm {log}}I_{i,t} = \eta + \varphi _1{\rm {log}}P_{i,t} + \varphi _2{\rm {log}}A_{i,t} + \varphi _3{\rm {log}}T_{i,t} + \mu _{i,t}$$

The extension of Eq. (2,) comprising covariate variables (*X*), can be written as Eq. (4), as follows:

4 $${\rm {log}}I_{i,t} = \varphi _0 + \varphi _1{\rm {log}}P_{i,t} + \varphi _2{\rm {log}}A_{i,t} + \varphi _3{\rm {log}}T_{i,t} + \beta {\rm {log}}X_{i,t} + s_i + \mu _{i,t}$$

In Eq. (4), *s* and *μ* represent unobserved factors of the error term, which have a normal distribution.

The covariate variables in this study were renewable energy deployment and inflation rates, which impact climate change.

Data on the variables (carbon emissions per capita, labor force, income per capita, green finance, inflation, and renewable energy deployment) were gathered from 2010 to 2021 for 25 Chinese provinces. The data were collected from China’s National Bureau of Statistics, the Statistical Yearbook of each province, and the China Statistical Yearbook on the environment. It should be noted that China’s first green bonds were printed in 2015, which is why we extracted the green financing variable of Chinese provinces since 2010 using the weighted average of green loans and green bonds.

To estimate the coefficients in Eq. (4), the system GMM approach proposed by Arellano and Bover (1995) is employed. The empirical model, in the form of the system GMM approach, is shown in Eq. (5) as follows:

5 $$y_{i,t} = \alpha y_{i,t - 1} + \beta ^\prime X_{i,t} + \mu _i + \varepsilon _{i,t}$$

where *y* is the dependent variable, *y*_*t*−1_ represents the lag term, and *X* represents the independent variables. It should be mentioned that the before–after estimation tests are conducted to explore the appropriate estimation technique and ensure the validation of the empirical findings.

## Empirical results

In the first stage, it is necessary to perform tests to determine the most appropriate estimation technique. Accordingly, a first-generation panel unit root test (ADF test) is conducted. Table 2 reports the results of the stationarity test of the series.

**Table 2:** First-generation panel unit root test.

Variable	ADF stat.	Probability
Carbon emissions per capita	−1.743	0.045
Labor force	−3.488	0.000
Income per capita	−2.944	0.024
Green finance	−3.100	0.000
Consumer price index	−5.883	0.015
Renewable energy deployment	−2.943	0.049

Source: The authors.

It is argued that, in panel data studies, there may be cross-sectional dependency among the samples; therefore, it was necessary to carry out the second generation of panel unit root tests (CIPS and C.Z. tests). The results of the two tests are presented in Table 3.

**Table 3:** Second-generation panel unit root tests.

Variable	CIPS	Probability	C.Z.	Probability
Carbon emissions per capita	−2.554	0.013	−1.232	0.014
Labor force	−2.821	0.003	−2.493	0.025
Income per capita	−3.193	0.030	−2.044	0.019
Green finance	−2.442	0.014	−1.483	0.015
Consumer price index	−2.593	0.041	−3.830	0.010
Renewable energy deployment	−2.993	0.039	−2.391	0.024

Source: The authors.

Based on the results of the first- and second-generation panel unit root tests, it can be concluded that all variables were stationary and stable processes.

Next, the coefficients were estimated using the system GMM technique. The results of the estimations are presented in Table 4.

**Table 4:** System GMM estimations for all examined provinces in China.

Variable	Coefficient	*t*-stat	*p*-value
Lagged CO_2_ emissions per capita	0.329	3.10	0.074
Labor force	0.013	2.54	0.031
Income per capita	0.033	3.92	0.002
Green finance	−0.015	−3.10	0.063
Consumer price index	0.049	1.93	0.002
Renewable energy deployment	−0.103	−3.04	0.008
Sargan test	0.9541		
AR (1)	0.0005		
AR (2)	0.2943		

Source: The authors.

Table 4 shows that carbon dioxide emission per capita positively affects this variable in the present time, and this effect was statistically significant. The labor force has a positive coefficient, meaning that a 1% increase in China’s labor force leads to increased carbon dioxide emissions per capita. An increase in the labor force in a country will mean an increase in industrial production capacity and, subsequently, in the country’s economic growth. In a country like China, whose manufacturing industries depend on fossil fuels, the increase in economic growth from the growth of industrial production will result in more carbon dioxide emissions. China’s per capita income also positively affects carbon dioxide emissions. An increase in per capita income means an increase in people’s purchasing power and consumption of more goods. This consequently suggests an increase in fossil energy consumption and carbon dioxide emissions. Green financing has a small, negative impact coefficient. By increasing the volume of green financing in China, carbon dioxide emissions can decrease by 0.015%. The consumer price index (CPI) positively and significantly affects carbon dioxide emissions. Generally, with an increase in the CPI, the capital rental rate and acquisition of bank facilities increase; therefore, the price of green projects increases. As a result, investors will not desire to participate in such projects; therefore, the excessive consumption of fossil fuels will continue in China, causing more carbon dioxide emissions. Renewable energy consumption had a coefficient of 0.103. Therefore, a 1% increase in renewable energy consumption is expected to decrease CO_2_ emissions by 0.103%.

Given the vast geographical area of China and the difference in the level of development across its provinces, the effectiveness of green financing and consumption of renewable energy varies from province to province. To obtain more practical findings, we categorized 25 selected provinces based on their geographical location into the following three regions: west, east, and center. The three-panel frameworks for these three regions were analyzed using the system GMM, as reported in Table 5.

**Table 5:** System GMM estimations for three regions of China.

Variable	Central region	Eastern region	Western region
Lagged CO_2_ emissions per capita	0.345 (0.01)	0.003 (0.449)	0.132 (0.002)
Labor force	0.409 (0.00)	0.023 (0.032)	0.033 (0.000)
Income per capita	0.392 (0.00)	−0.112 (0.000)	0.119 (0.00)
Green finance	−0.001 (0.392)	−0.422(0.01)	−0.031 (0.00)
Consumer price index	0.312 (0.00)	0.132 (0.00)	0.178 (0.042)
Renewable energy deployment	−0.003 (0.00)	−0.221 (0.04)	−0.004 (0.023)
Sargan test	0.853	0.911	0.983
AR (1)	0.015	0.019	0.003
AR (2)	0.483	0.432	0.231

Numbers in () are *p*-values.

Source: The authors.

Lagged CO_2_ emissions impacted only the provinces in the central and western regions of China, while the coefficient of lagged CO_2_ emissions was not statistically significant for the eastern region’s provinces. The labor force across all three regions of China positively and significantly affected carbon dioxide emissions. The magnitude of this effect in the central region was greater than that in the other two regions, a major reason for which is the existence of many heavy industries in the provinces of the central region of China. Regarding income per capita, the estimation results showed that the effect of this variable on carbon dioxide emissions was positive in the central and western regions, and only the states located in the eastern region of China had a negative effect coefficient. This may be because the eastern provinces of China are more developed, and the higher the income levels in these provinces, the more the people are willing to change their lifestyles in a green way to protect the environment. Green financing had a statistically significant coefficient only in provinces in the eastern and western regions. Moreover, this variable’s influence on reducing carbon dioxide emissions was more significant in the eastern region of China. An important reason for this is that the eastern regions of China are more developed. Therefore, people in these regions have greater potential to participate in the green financing market and green projects. The use of renewable energy across all regions of China has had a positive effect on reducing carbon dioxide emissions. However, its influence in the eastern region has been greater than that in the other two regions. The CPI also had a significant coefficient and positive sign for all three regions. Therefore, in all provinces of China, an increase in the CPI is an obstacle to developing sustainable development policies.

To ascertain the validity of the empirical findings, a robustness check was employed. Accordingly, the dependent variable (CO_2_ emissions per capita) was substituted using greenhouse gas emissions. The empirical model was re-estimated using the system GMM approach for Chinese regions. The results, as reported in Table 6, confirmed that the signs of the coefficients were consistent with the earlier estimations (Table 5).

**Table 6:** Robustness check.

Variable	Central region	Eastern region	Western region
Lagged greenhouse gas emissions	0.019 (0.04)	0.13 (0.166)	0.203 (0.00)
Labor force	0.011 (0.08)	0.281(0.00)	0.113 (0.04)
Income per capita	0.025 (0.01)	−0.014 (0.00)	0.202 (0.06)
Green finance	−0.13 (0.332)	−0.109(0.05)	−0.094 (0.01)
Consumer price index	0.114 (0.04)	0.54 (0.01)	0.039 (0.023)
Renewable energy deployment	−0.013 (0.02)	−0.522 (0.02)	−0.032 (0.013)

Numbers in () are *p*-values.

Source: The authors.

## Conclusions and policy recommendations

### Concluding remarks

The threat of climate change has become a topic of concern for the international community in recent decades. Despite several meetings and discussions with experts and politicians, humankind has not been able to remove the shadow of this threat from life on the planet. Given its high volume of fossil fuel use, China has made a significant contribution to the creation and persistence of this threat. In this study, using data from 2010 to 2021, the STIRPAT theory, and the econometric method of panel data, the effect of green financing and deployment of renewable energy on the carbon dioxide emissions of China and its provinces have been analyzed and interpreted. The findings show that green financing has a slight negative impact coefficient on environmental pollution at the country level. By increasing the volume of green financing in China, carbon dioxide emissions can decrease by 0.015%. Moreover, renewable energy consumption has a coefficient of 0.103. This indicates that by increasing renewable energy consumption by 1%, CO_2_ emissions can be expected to decrease by 0.103%. However, at the regional level of analysis, green financing has a statistically significant coefficient only for the provinces located in the eastern and western regions. Additionally, this variable’s influence on reducing carbon dioxide emissions is more significant in the eastern region of China. The critical reason for this is that the eastern regions of China (e.g., Shanghai) are more developed; therefore, the people in these regions have more potential to participate in the green financing market and green projects. The use of renewable energy across all regions of China has had a positive effect on reducing carbon dioxide emissions. However, the influence in the eastern region has been greater than that in the other two regions.

### Practical policies

According to the obtained results, the issue of clean energy policies at the provincial and country levels in China is worth mentioning. The Chinese government should pay more attention to encouraging leading provinces in the field of renewable energy and supporting lagging provinces in the fight against environmental pollution. Chinese leaders must make strong decisions about the future of their country by supporting sustainable development. On September 22, 2020, the president of China announced that, in line with the Sustainable Development Goals, his country would try to reach peak emissions before 2030 and carbon neutrality before 2060. To achieve these two important targets, China should formulate and approve two separate policy groups at the national and provincial levels. At the macroeconomic level, China’s policymakers should try to create green jobs (to create a positive effect where increasing the labor force reduces carbon dioxide emissions), increasing the green basket of consumption in society (increasing the per capita income will improve environmental conditions), better development of the green financing market, wider financial support for green projects (to neutralize the negative effect of inflation on the development of green projects), and make more efforts to increase the consumption of renewable energy. However, at the regional and provincial levels, China’s policymakers should institute more incentive policies for the provinces in the eastern region so that they can move more easily along the path of sustainable development. Conversely, the central region should be under supportive policies (granting green loans and green subsidies) and pressure policies (carbon tax) to move faster toward sustainable development. The uniformity of applied policies toward different provinces and regions can become a factor in their ineffectiveness and the creation of inequality in the sustainable development of regions in the long term (Chen, 2022).

### Future research recommendations

This study provides practical findings and innovative management policies for experts and policymakers in China and worldwide. It is important that China, as the largest contributor of greenhouse gas emissions and a major consumer of fossil fuels, creates a managed balance in its provinces in the field of sustainable development. The results of this study significantly contribute to achieving this goal. However, there were limitations (e.g., access to local data) in conducting this research. It is suggested that, in future research, field research and survey methods be used to obtain complementary results. Given the spread of COVID-19 since the end of 2019 and its transformation into a worldwide pandemic, future research should investigate the effect of this pandemic on the relationship between green financing and climate change. More advanced econometric methods, such as artificial neural networks, could be used to predict the relationships between variables in the future. Such research can help China reach its goals of peak carbon by 2030 and carbon neutrality by 2060.

## Data Availability

The datasets generated and analyzed in the current study are available from the corresponding author upon reasonable request.
